# Clinical evaluation of nebulized verapamil in out‐patients with pulmonary hypertension secondary to chronic obstructive pulmonary disease

**DOI:** 10.1111/crj.13551

**Published:** 2022-11-06

**Authors:** Fanak Fahimi, Guitti Pourdowlat, Neda Behzadnia, Sahar Sadigh Mostofi, Aida Sefidani Forough, Omid Parto, Ayda Esmaeili

**Affiliations:** ^1^ American Career College Anaheim USA; ^2^ Chronic Respiratory Disease Research Center, National Research Institute of Tuberculosis and Lung Diseases (NRITLD) Shahid Beheshti University of Medical Sciences Tehran Iran; ^3^ Clinical Pharmacy Department, School of Pharmacy Shahid Beheshti University of Medical Sciences Tehran Iran; ^4^ School of Clinical Sciences, Faculty of Health Queensland University of Technology (QUT) Brisbane Australia; ^5^ Concorde Career College Garden Grove California USA; ^6^ Experimental and Applied Pharmaceutical Sciences Research Center Urmia University of Medical Sciences Urmia Iran; ^7^ Department of Clinical Pharmacy, School of Pharmacy Urmia University of Medical Sciences Urmia Iran

**Keywords:** calcium channel blocker, COPD, inhaled verapamil, nebulization, pulmonary function test, pulmonary hypertension, spirometry

## Abstract

**Objective:**

Chronic obstructive pulmonary disease (COPD) is associated with many health complications, including pulmonary hypertension (PH). Although oral calcium channel blockers have shown promising results in managing COPD‐induced PH, significant systemic side effects may limit their use in this population. Administering verapamil through nebulization can be an alternative approach. We aim to assess the possible therapeutic effects of verapamil inhalation in out‐patients with pulmonary hypertension (PH) secondary to COPD.

**Methods:**

A double‐blind, randomized placebo‐controlled clinical trial was conducted. Patients with PH were randomly assigned to two groups of 15 participants. The intervention group received a short‐term single dose of 10 mg nebulized verapamil (4 ampoules of 2.5 mg/ml verapamil solutions). The control group received nebulized distilled water as a placebo in addition to their standard treatment throughout the study.

**Results:**

Systolic pulmonary artery pressure (sPAP) did not improve as a primary outcome significantly in patients receiving nebulized verapamil compared with those on placebo (*p* = 0.89). Spirometry results showed a significant improvement in FVC in the intervention group from 1.72 ± 0.63 to 1.85 ± 0.58 L (*p* = 0.00), and FEV1/FVC ratio decreased significantly after verapamil administration (*p* = 0.027).

**Conclusion:**

Verapamil did not improve any of the pulmonary artery or RV parameters in patients with COPD‐associated, but it did improve SpO_2_ and increase FVC, which revealed us possibility of verapamil in treating V/Q mismatch. The improved gas exchange may have been due to improvements in FVC as reflected in the improved spirometry. Higher doses of verapamil may be more efficacious and can be the subject of future trials.

## Graphical abstract

Single dose nebulized verapamil inhalation showed improvement in V/Q mismatch throught increase FVC. Nebulized verapamil inhalation improved gas exchanged and increased spO2. Nebulized verapamil inhalation for out‐patients with pulmonary hypertension secondary to chronic obstructive pulmonary disease improved spirometric parameter. The FEV1/FVC ratio decreased significantly after verapamil administration due to more potent bronchodilator on small airways.

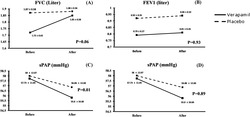

## 1 INTRODUCTION

Chronic obstructive pulmonary disease (COPD) is associated with many health complications, among which pulmonary hypertension (PH) is a serious life‐threatening condition. Patients with COPD usually present with mild to moderate PH. PH chiefly results from severe limitation in expiratory airflow and the consequent hypoxia. The aetiologies of PH, including pathologic changes and most commonly vascular remodelling in pulmonary arterioles, are well‐known.^1^, ^2^ Pulmonary hypertension is one of important indicators of the prognosis of COPD and survival among hospitalized patients.^3^, ^4^ Prevalence of PH has shown to be ranging from 50% of patients with moderate COPD to 70%–90% in severe COPD cases.^5^, ^6^, ^7^, ^8^ The mechanism by which obstructive airway disease causes PH lies in the association of pulmonary arterial pressure (PAP) with three major determinants of cardiac output (CO), pulmonary vascular resistance (PVR), and pulmonary arterial wedge pressure (PAWP). The air trapped in the lungs during physical activity causes increased PVR and PAWP and consequently increases PAP due to elevated resistance.^9^, ^10^ As a result of this, the PAP may increase remarkably during exercise, sleep, and exacerbation episodes, reaching twice as high as the baseline pressure at times.^11^

A range of treatments is known for COPD‐related PH, including oxygen therapy, general and specific vasodilators, statins, phlebotomy, lung volume reduction surgery (LVRS), and even lung transplantation.^12^, ^13^, ^14^, ^15^, ^16^, ^17^, ^18^ Long‐term oxygen therapy is one of the essential treatments for the condition, whereas systemic medications are not commonly administrated due to the risk of ventilation/perfusion (V/Q) mismatch they can cause in patients. Advanced therapy options include prostacyclin pathway agonists, endothelin receptor antagonists, nitric oxide (NO)‐cGMP enhancers, and calcium channel blockers. It is recommended that patients who have PH due to lung disease and/or hypoxaemia should not undergo advanced therapy unless referred to a specialized centre with skilled pulmonologists for evaluation; vasodilators such as calcium channel blockers (CCBs), β2‐agonists, nitrates, angiotensin‐converting enzyme inhibitors, theophylline, and α1‐antagonists decrease the pressure and vascular resistance in PH. However, side effects such as systemic hypotension, worsening of pulmonary gas exchange, and aggravation of V/Q mismatch limit their broad systemic use in long‐term trials.^19^, ^20^, ^21^, ^22^

For years, CCBs remained the only oral drug class available for pulmonary hypertension.^23^ They have been studied in COPD‐associated PH with promising results such as improved dyspnoea.^24^, ^25^ The systemic nifedipine is the most studied medication of this class for PH indication.^21^, ^26^, ^27^, ^28^ However, nifedipine causes a significant systemic hypotensive effect, which is more detrimental if the pulmonary artery is hyporeactive to its vasodilatory effect. Also, the sympathetic tone may be activated in response to the decreased blood pressure and lead to more intense pulmonary hypertension. On the other hand, peripheral oedema is another common side effect of dihydropyridine CCBs like nifedipine^29^ and thus could be misdiagnosed as a sign of right heart failure.

Although the use of non‐dihydropyridine CCBs like verapamil has been discouraged in PH due to its negative inotropic effect,^30^, ^31^ it can theoretically exert preferential impact on the extremely constricted specific vascular beds, that is, pulmonary, by blocking calcium channels. Inhalation, as an alternative to oral drug delivery, is a usual route of drug administration in many respiratory disorders. Verapamil inhalation can produce higher concentrations in the airway with the least systemic effects as well as avoiding first‐pass hepatic metabolism. Verapamil, used in inhaled form, is naturally expected to reach more expansive areas with better ventilation, and it is expected to have a lower risk of V/Q mismatch. The current study hypothesized that inhaling verapamil allows more rapid achievement of therapeutic effect while avoiding V/Q mismatch and systemic side effects such as hypotension. Therefore, it aimed to investigate the clinical effect of nebulized verapamil in the management of COPD complication especially pulmonary hypertension.

## 2 MATERIALS AND METHODS

### 2.1 Study design

This study was a double‐blind randomized placebo‐controlled clinical trial (RCT), registered at the Australian New Zealand Clinical Trials Registry, which can be accessed via www.anzctr.org.au (registration number: ACTRN12612001239853).

### 2.2 Participants and inclusion/exclusion criteria

Participants were patients with pulmonary hypertension secondary to COPD, referred to a tertiary university hospital in Tehran, Iran, between February 2012 and December 2012. Patients who fell under the group III according to 6th World Symposium on Pulmonary Hypertension (WSPH) were eligible for inclusion. According to the WSPH classification, different types of PH are categorized based on the cause of the disease, among which group III pertains to PH due to lung disease and/or chronic hypoxia. Patients were considered for inclusion if they presented with moderate severity of PH (PAP ≥ 45 mmHg) as those with mild level PH generally do not require immediate treatment. In addition to this major criterion, patients were eligible for inclusion in the trial if they had normal left ventricular ejection fraction in echocardiography (EF > 55%). The exclusion criteria were recent respiratory infection and sepsis, end‐stage renal and hepatic dysfunction, extensive burn, pregnancy, arrhythmia (atrial fibrillation/flutter with auxiliary bypass track like Wolff–Parkinson–White and Lown–Ganong–Levine), grade II and III of the cardiac block, drop EF to less than 40% during the study, and COPD exacerbation. Moreover, patients with a history of pulmonary embolism, CHF, and sleep apnoea were excluded due to their additional possible causality on PH. The medical charts were reviewed in pulmonology and cardiology wards, emergency room, and CCU to assess patients' eligibility for the study. Patients were then enrolled in the study if they met the inclusion criteria. Demographic information such as age, gender, height, weight, duration of disease, and New York Heart Association functional classification in pulmonary hypertension were recorded.

### 2.3 Measurements

Diagnosis of COPD in patients was reliably established by a pulmonologist (G. P.). The diagnosis was confirmed by a cardiologist (N. B.) who also carried out transthoracic echocardiography before and during the study for each patient. Echocardiography was performed to assess ejection fraction (EF), systolic pulmonary arterial pressure (sPAP), right ventricular (RV) size, and RV TAPSE (tricuspid annular plane systolic excursion). Baseline pulmonary function tests, including spirometry and measurement of forced expiratory volume in 1 s (FEV1), forced vital capacity (FVC), and FEV1/FVC were also carried out and 15 min after intervention. Oxygen saturation (SpO_2_) was measured by pulse oximetry before and 15 min after the intervention in both groups, three times with 5 min intervals in each section to get the mean reading. Blood pressure and heart rate were also monitored for evaluation of the intervention's possible side effects.

### 2.4 Randomization

Participants were randomly divided into two groups of intervention and placebo, based on computer‐generated random codes. The investigators and the patients were blind to the medication. The medications were prepared and coded by the clinical pharmacist (F. F.) at the time of use to prevent any information leak from interfering with the blindness of the study.

### 2.5 Data collection

Demographics including age, sex, height, and weight in addition to past medical history were initially recorded from the patients' medical sheets. Clinical information was gathered after the randomization. Blood pressure, heart rate, SpO_2_, echocardiography, and spirometry findings were recorded before and 15 min after the intervention.

### 2.6 Intervention

People in the intervention group received single dose nebulized verapamil inhalation at the dose of 10 mg with the final concentration of 2.5 mg/ml by Ultrasonic Nebulizer CUN60 (CITIZEN®, Japan). This dose was selected according to the previous studies and considering the possibility of bronchospasm in doses higher than 10 mg.^32^ Verapamil was prepared in the ampule form named Lekoptin® (Lek Pharmaceutical Company dd, Slovenia, the enterprise of Sandoz company) with the pH = 4–6.5. The control group was administered distilled water as a placebo, throughout the study while receiving their usual standard treatment.

The water reservoir of the nebulizer device was filled at room temperature. The medication was placed in a particular container and then in the water tank. Ten to 15 min were required to complete the nebulization of 4 ml (10 mg) verapamil for the intervention or 4 ml distilled water for the placebo group.

Nebulization stopped if a patient complained of any side effects, for example, vertigo, hypotension, bradycardia, nausea, and headache according to the study protocol. Also, the intervention was stopped in case of dyspnoea and a fall in SpO_2_, needing O_2_ therapy.

### 2.7 Outcome measures

A 20% or more reduction in PAP as main out come and improved at least 12% improvement in FEV1 and/or FVC and ≥3% elevation in SpO_2_ after nebulization according to NYHA were considered as a positive response to verapamil inhalation treatment. The PAP was measured using a colour ultrasonic Doppler echocardiography under the CDFIC21 (by Jiangsu Jiahua®, China). Fingertip pulse oximeter Prince‐100 (by Shenzhen Creative ®, China) was utilized for measuring SpO_2_ and pulse rate in both intervention and control groups. Spiro lab® (Italy) pulmonary function test was deployed three times for each patient to ensure the reliability of spirometry readings.

### 2.8 Statistical analysis

SPSS statistics software version 16.0 was used for all statistical analyses. Normal distribution of data was tested before the selection of the proper statistical tests, enabling the use of parametric statistics such as a one‐sample *t*‐test. When the population could not be assumed to be normally distributed, the Mann–Whitney *U* test as a non‐parametric statistical hypothesis test was used for independent samples. Pearson's *χ* ^2^ test was used for the analysis of categorical data. A paired *t*‐test was used to compare the results before and after the intervention. Data were expressed as mean ± SD. A *p*‐value of <0.05 was considered statistically significant in all of the cases.

### 2.9 Ethical considerations

Verapamil was administered under close monitoring by a healthcare team for observing and preventing any suspected side effects of inhaled verapamil, which could result in the discontinuation of the trial. All participants were provided with a comprehensive description of the aims, process, and possible benefits and risks of the study before giving their written informed consents. There was no extra charge for the patients who participated in this study and all the information was collected and stored securely by the investigators. The participants were able to withdraw from the study whenever they wished without comment or penalty.

## 3 RESULTS

**FIGURE 1:**
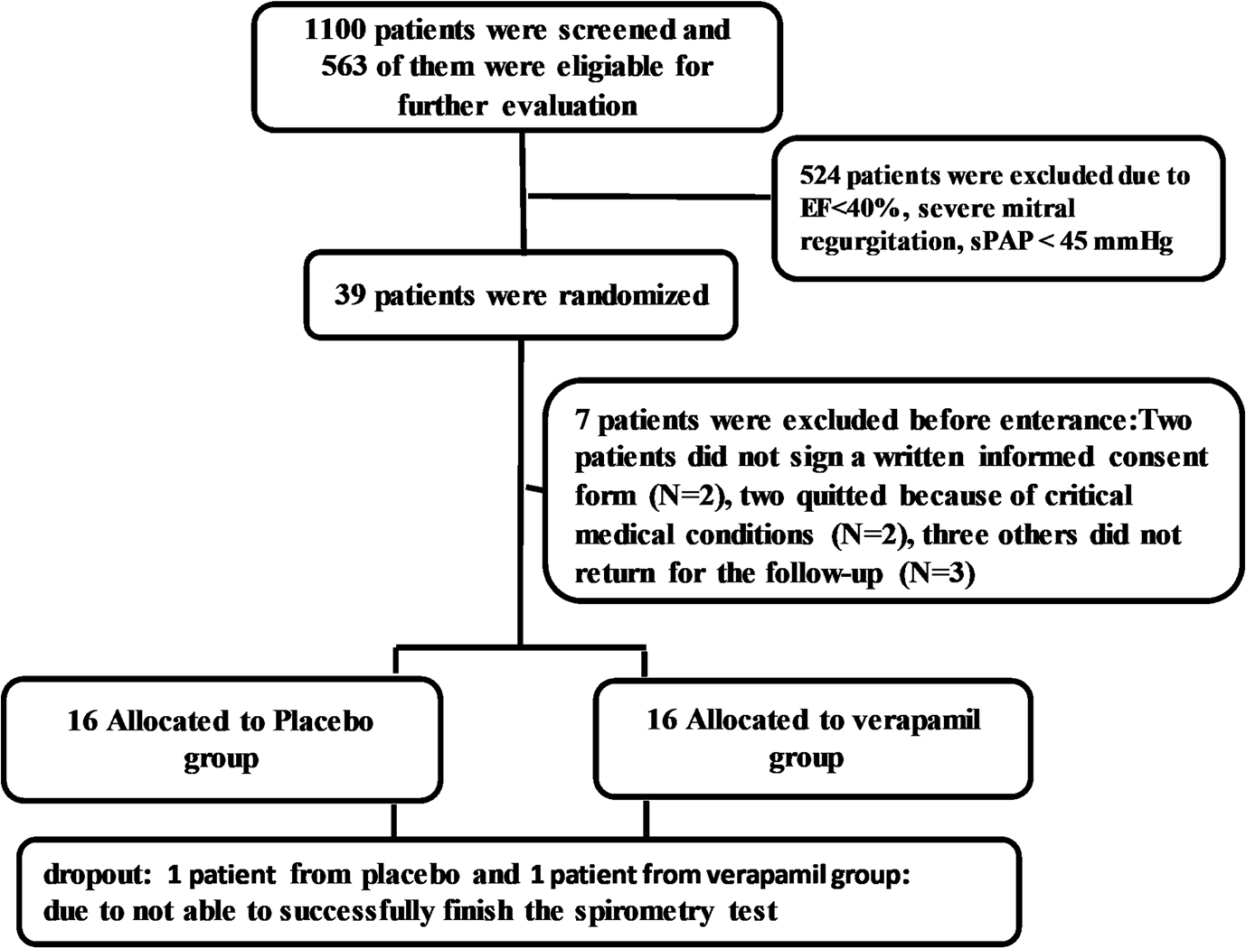
The flowchart of the study

This study evaluated 1100 records for COPD patients, among which 563 had echocardiography and examination reports available. Thirty‐nine patients eventually met the inclusion criteria. Two patients had no interest in participating in the trial, and two quitted because of critical medical conditions. Three others did not return for the follow‐up clinical tests, and two were not able to successfully finish the spirometry test. The flow chart is presented these data (Figure 1).

Thirty patients were eventually recruited and divided into two groups of verapamil nebulization (treatment) and placebo group. The male/female ratio was 26/4.

No significant difference was observed in age, sex distribution, duration of the disease, and body mass index between the placebo and verapamil groups (Table 1).

**TABLE 1:** Demographic data of the participants in verapamil and placebo arm

Group variable	Verapamil	Placebo	*p*‐value
Gender	Female 1 (6.7%)	Female 3 (20%)	0.598
	Male 14 (93.3%)	Male 12 (80%)	
Age (years)	63.40 ± 7.60	59.73 ± 9.20	0.244
BMI	23.94 ± 6.09	26.67 ± 4.85	0.184
Duration of disease (years)	6.53 ± 4.08	6.13 ± 5.23	0.817
SpO_2_%	77.87 ± 9.94	78.07 ± 11.74	0.233
sPAP, mmHg	57.73 ± 11.05	58.00 ± 12.07	0.375
EF	52.67 ± 2.58	55.00 ± 4.23	0.04
Functional classification	III 9 (60%)	III 11 (73.3%)	0.700
	IV 6 (40%)	IV 4 (26.7%)	

The mean duration of COPD diagnosis was 6.33 ± 4.65 years, ranging from 1 to 15 years.

The differences between EF, systolic PAP, RV size, RV TAPSE, SpO_2_, and spirometric values (FEV1, FVC, and FEV1/FVC) before and after the intervention in the placebo group were not statistically meaningful (*p* > 0.05).

**FIGURE 2:**
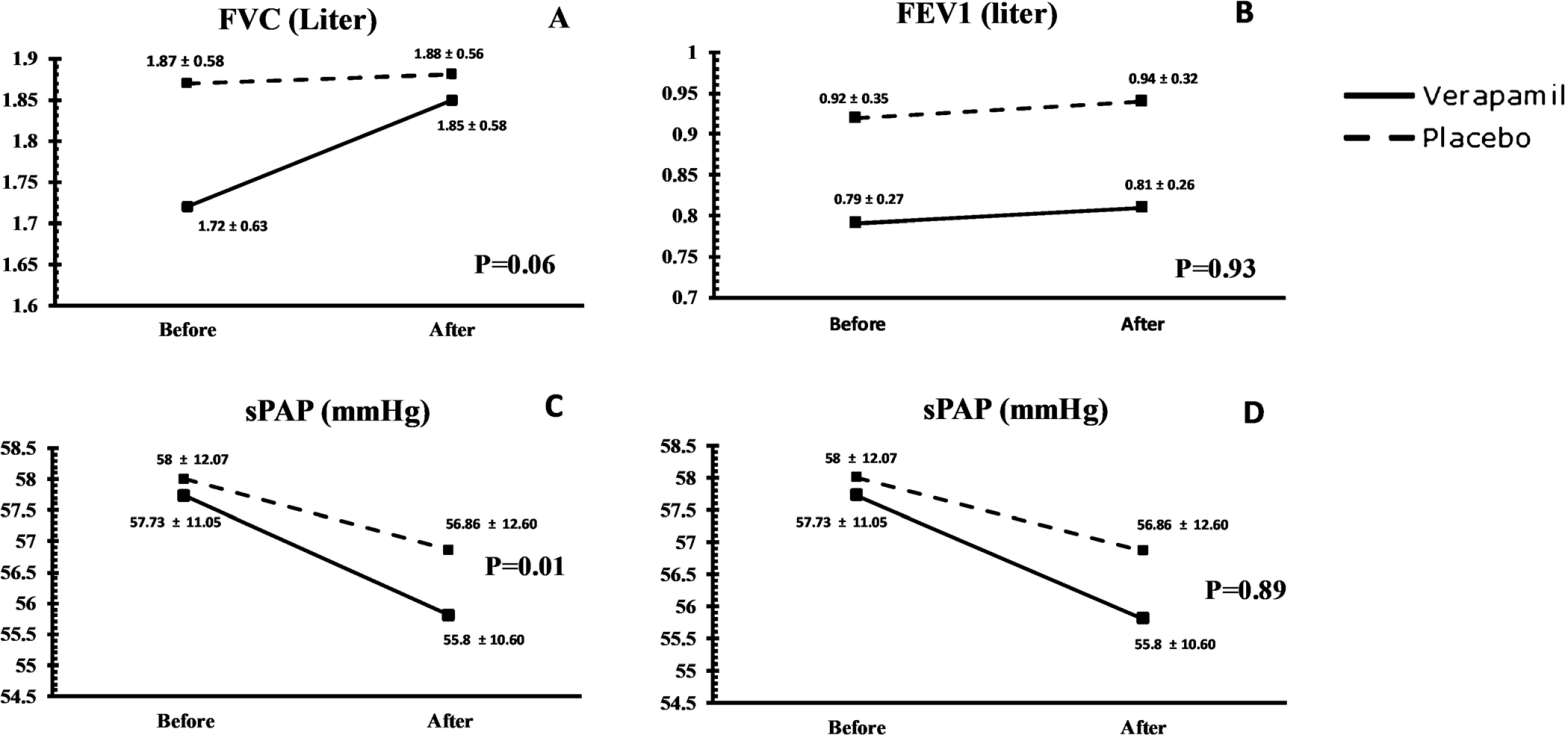
Effects of inhaled verapamil and distilled water on FVC (%), FEV1/FVC (%), SpO_2_ (%), and sPAP are shows in a, B, C and D, respectively. FEV1, first second of forced expiration; FVC, forced vital capacity; sPAP, systolic pulmonary artery pressure

In the intervention group, inhalation of verapamil led to a significant increase in the mean SpO_2_ level from 77.87% ± 9.94% before the test to 81.00% ± 10.09% after the test (*p* = 0.01). Similarly, FVC values after the verapamil inhalation were improved significantly from 1.72 ± 0.63 to 1.85 ± 0.58 L (*p* = 0.00). However, when compared with the placebo group, this increase in FVC was not statistically meaningful (*p* = 0.08). Notably, none of the remaining parameters, such as EF, systolic PAP, RV size, and RV TAPSE were associated with significant improvements. The results of the before and after respiratory parameters changes in the verapamil group are presented in Figure 2.

In the verapamil group, significantly more patients self‐reported subjective improvement in the transient respiratory symptoms compared with the placebo group. Table 2 demonstrates the differences of parameters between the placebo and verapamil groups. There was a significant improvement in SpO_2_ levels after verapamil administration (*p* < 0.001) and also compared with the placebo group (*p* = 0.01). The changes in FEV1/FVC ratio also differed significantly between the placebo and the intervention group (*p* = 0.03). FEV1/FVC ratio decreased substantially in the verapamil group compared with the placebo group (*p* = 0.04).

**TABLE 2:** Difference in changes between verapamil and placebo arm

s	Medicine	*N*	Mean ± std. deviation of difference	*p*‐value
EF	Verapamil	15	0.00 ± 0.00	1.00
	Placebo	15	0.00 ± 0.00	
sPAP	Verapamil	15	−1.93 ± 4.32	0.89
	Placebo	15	−1.13 ± 2.80	
RV size	Verapamil	15	−0.03 ± 0.22	0.83
	Placebo	15	−0.05 ± 0.22	
RV TAPSE	Verapamil	15	0.43 ± 2.48	0.14
	Placebo	15	−0.80 ± 1.89	
FEV_1_ (actual) (L)	Verapamil	15	−0.02 ± 0.06	0.93
	Placebo	15	−0.02 ± 0.09	
FVC (actual) (L)	Verapamil	15	−0.14 ± 0.15	0.06
	Placebo	15	0.03 ± 0.14	
FEV_1_/FVC ratio	Verapamil	15	−2.88 ± 4.53	0.04
	Placebo	15	0.21 ± 3.03	
SpO_2_	Verapamil	15	−3.13 ± 2.59	0.01
	Placebo	15	−0.53 ± 2.17	

Abbreviations: EF, ejection fraction; sPAP, systolic pulmonary artery pressure; RV, right ventricular; TAPSE, tricuspid annular plane systolic excursion; FEV1, first second of forced expiration; FVC, forced vital capacity.

Inhalation of verapamil did not show significant changes in cardiac parameters nor did it cause any side effects such as systemic hypotension or bradycardia during or after the intervention. EF remained constant in all groups with no considerable change. The SV/EDV and ESV/EDV ratios were also stayed unchanged in both groups indicating that SV has remained constant. Patients' heart rate also remained stable in both groups, and consequently, cardiac output and cardiac index also remained unchanged before and after the intervention. These findings indicate no effect for 10 mg inhaler verapamil on pulmonary arterial pressure (PAP) in COPD patients and no significant change in echocardiographic results in each group and between two groups before and after the intervention. Measurements of heart rate, systolic blood pressure, and diastolic blood pressure, before and after the test are presented in Table 3.

**TABLE 3:** Adverse events verapamil and placebo arm

	Placebo			Verapamil		
	Before	After	*p*‐value	Before	After	*p*‐value
Diastolic BP (mmHg)	75 ± 9	75 ± 8	0.72	71 ± 9	73 ± 10	0.14
Systolic BP (mmHg)	121 ± 19	121 ± 19	0.82	116 ± 18	117 ± 19	0.40
Heart rate (/min)	87 ± 16	87 ± 13	0.37	94 ± 26	94 ± 24	0.61

## 4 DISCUSSION

This study assessed the therapeutic effect of inhaled verapamil in patients with COPD‐induced pulmonary hypertension. Although all patients continued receiving their standard treatment for PH, participants in the intervention group were administered an additional dose of inhaled verapamil. To the best of the authors' knowledge, this study is the first trial to assess the therapeutic effects of inhaled verapamil on PH secondary to COPD in outpatients. There are, however, reports available in the literature which have investigated the effectiveness of other forms of verapamil such as injection on PH. In summary, the result of this study showed that the nebulized verapamil in patients with secondary pulmonary hypertension due to COPD could not improve sPAP.

In 1978, Landmark and colleagues injected verapamil into the pulmonary artery in 12 PH patients. Contrary to non‐significant cardiac changes in our study, the verapamil injection resulted in reduced CO and PVR in 3/12 patients, although these changes were not statistically significant.^33^ The difference between the findings may address the different effects of inhaled and injective verapamil in this regard. Different from dihydropyridine calcium channel blockers like nifedipine which are most effective on vessels to dilate peripheral and coronary system, non‐dihydropyridine calcium channel blockers such as verapamil directly affect the heart to reduce heart rate and cardiac contraction, inhibit atrioventricular node (AV node), and rather increase in coronary blood flow. With considering the cardiac output as a function of HR and SV, verapamil injection can result in a drop in CO and consequently CI, by reducing heart rate. It seems that the negative effects are not expected with the 10 mg inhaled form of verapamil as these effects were not observed with the used dose in the current study. This route of use may provide a benefit and a promising alternative for verapamil administration in patients with COPD.

Few studies have reported on the effect of calcium channel blockers on the spirometry parameters. Expectedly, in this study, no significant changes were found in the placebo group in terms of FEV1, FVC, and FEV1/FVC after the study was completed compared with starting time. However, increase in FEV1 and FVC was observed in verapamil group; FVC increased significantly after using inhaled verapamil. On the contrary, no significant change was observed in FEV1. Due to more improvement in FVC, the FEV1/FVC ratio decrease significantly. This ratio does not appear to have any clinical implications and just misperception because more improvement of FVC led to decrease in FEV to FVC ratio. A previous double‐blinded trial found similar results to ours when assessed the effect of 20 mg inhaled verapamil on 15 bronchial asthma patients. Although non‐significant, increased FVC and PEF beside significant decreased pulmonary airway resistance (PAR) in their study indicated predominant bronchodilator effect of verapamil on large airways.^34^ according to more changes in FVC than FEV1, it is supposed that inhaled 10 mg verapamil dilated bronchioles more than bronchus, which potency bronchodilatory effect on respiratory system may have correlation with doses and frequency of administration, so need more investigations.

Inhaled nifedipine and verapamil with 5 mg dosage have been previously trailed in patients with bronchial asthma. They have shown significant changes in FVC and peak expiratory flow rate (PEFR) after 90–120 min from the administration.^35^

Verapamil injection in two other studies has also resulted in decreased PVR which is following this study's findings.^31^, ^36^ Verapamil injection resulted in decreased CI in some patients, which is not ideal in patients with COPD due to decreased fraction of inspired oxygen FiO_2_.

Arterial O_2_ saturation did not change throughout the study in the placebo group, whereas verapamil administration improved it significantly. This is chiefly resulted by inhaled verapamil because the inhaled medication could only flow through well‐ventilated alveolar parts of lung tissue and finally had the best vasodilatory effects. COPD generally causes vasoconstriction due to hypoxia in pre‐capillary and arterial beds. Systemic vasodilators increase blood flow in all vessels, even in pulmonary alveoli, with inadequate ventilation resulting in increased shunt and V/Q mismatch that worsen hypoxia. Inhalation, contrariwise, delivers the medication exactly to the alveoli with good ventilation to reduce V/Q mismatch in addition to improve blood oxygenation.^37^

The other hypothesis is that V/Q improved because of improved ventilation through improving small airways dilation, which could also have allowed an increase in FVC.

So it is desired to design further study in order to evaluate inhaled form of CCB in improving ventilation and reducing V/Q mismatch.

Side effects like vertigo, palpitation, and other subjective problems were recorded in this phase in addition to medical examination findings such as blood pressure, heart rate, and so on. The satisfaction rate was significantly higher in the intervention group than the placebo group due to the alleviation of respiratory symptoms the side effects were absent in both groups.

This study was subject to some limitations. Some COPD patients are either in an unstable condition or have comorbidities in cold seasons which make them too unstable to participate in similar studies. They also refer to hospitals or clinics less frequently during warmer seasons and the current research faced a challenge in sampling in this sense; therefore, the study power may be not enough to rely on results. Furthermore, the majority of patients had been on medications that the study focused on, like Calcium channel blockers, amlodipine, and diltiazem that made our wash‐out procedure unable to run regarding ethical features. Spirometry was the other challenge because patients are usually unfamiliar with the test or functionally are not able to do it. The study was designed in COPD population who had PH, but according to the result, it was better to focus more on general COPD population, because improvement in NYHA and SpO_2_ and PFT was significant, whereas no significant reduction observed in PAP.

Also, the sPAP was assessed by Doppler echocardiography, which has lower accuracy than right heart catheterization.

In addition to above‐mentioned limitations, a significant weakness is that this was designed in a parallel group study; however, a cross‐over study would have been easy to do and have had greater statistical power for judgment.

The optimal dose of verapamil is not known yet and the current study used 10 mg of the medication to prevent serious side effects, which may not be the optimal dose for this purpose. The absence of side effects during this trial indicates an insufficient dose of verapamil. Calcium channel blockers need a response assessment before administration, including intravenous (IV) prostacyclin or IV adenosine or inhaled nitric oxide (NO) through right atrial catheterization. At least a 20% reduction in PAP would support CCBs administration in these patients. To sum up, inhaled verapamil more than 10 mg may be effective in V/Q mismatch improvement, but it should be used in smaller particles to increased membrane permeability in alveoli besides using lecithin w, which could make the effectiveness of verapamil optimal.

## 5 CONCLUSION

Inhalation 10 mg single dose of Verapamil did not improve any of the pulmonary artery or RV parameters in patients with COPD‐associated, but it did improve SpO_2_, which revealed us possibility of verapamil in treating V/Q mismatch. The improved gas exchange may have been due to improvements in airways function as reflected in the improved spirometry. Higher doses of verapamil may be more efficacious and can be the subject of future trials.

## CONFLICTS OF INTEREST

There is no conflict of interest to declare. The financial support of the study was offered by Shahid Beheshti University of Medical Sciences, and all authors' financial support given in order to complete the study or write the manuscript.

## ETHICS STATEMENT

The study was approved by the Ethics Committee of Shahid Beheshti University of Medical Sciences and registered at the Australian New Zealand Clinical Trials Registry, which can be accessed via www.anzctr.org.au (Registration number: ACTRN12612001239853). This study was conducted in accordance with the Declaration of Helsinki and followed all institutional and national guidelines, as well as regulations relevant to human experimentation.

## AUTHOR CONTRIBUTIONS

**Fanak Fahimi:** Conception and design of the study, the experiments and procedures, statistical analysis, preparation of manuscript draft, corrected and confirmed the final manuscript, corresponded. **Guitti Pourdowlat:** Conception and design of the study, Generation, collection, assembly, patient assessment, analysis and/or interpretation of data the experiments and procedures. **Ayda Esmaeili:** the experiments and procedures, Drafting or revision of the manuscript and rewriting of article. confirmed the final manuscript, **Neda Behzadnia:** The generation, collection, assembly, experiments and procedures, confirmed the final manuscript. **Sahar Sadigh Mostofi:** statistical analysis, the experiments and procedures. **Omid Parto:** The experiments and procedures, assisting in analysis, confirmed the final manuscript. **Aida Sefidani Forough:** The experiments and procedures, confirmed the final manuscript.

## Data Availability

The data that support the findings of this study are available on request from the corresponding author. The data are not publicly available due to privacy or ethical restrictions.
